# The Risk of Developing Osteoporosis in Hemolytic Anemia—What Aggravates the Bone Loss?

**DOI:** 10.3390/jcm10153364

**Published:** 2021-07-29

**Authors:** Leiyu Shi, Cheng-Li Lin, Ching-Huang Su, Keng-Chian Lin, Kam-Hang Leong, Yu-Ting Tina Wang, Chien-Feng Kuo, Shin-Yi Tsai

**Affiliations:** 1 Department of Health Policy and Management, Johns Hopkins University Bloomberg School of Public Health, Baltimore, MD 21205, USA; lshi2@jhu.edu; 2 College of Medicine, China Medical University, Taichung City 404, Taiwan; orangechengli@gmail.com; 3 Department of Laboratory Medicine, Mackay Memorial Hospital, Taipei City 104, Taiwan; u101001029@cmu.edu.tw (C.-H.S.); kerrapple@gmail.com (K.-C.L.); firzenleong625@gmail.com (K.-H.L.); tinaw3478@gmail.com (Y.-T.T.W.); 4 Division of Infectious Diseases, Department of Internal Medicine, Mackay Memorial Hospital, Taipei City 104, Taiwan; kakutsai1230@gmail.com; 5 Department of Medicine, Mackay Medical College, New Taipei City 25245, Taiwan; 6 Department of Cosmetic Applications and Management, MacKay Junior College of Medicine, Nursing and Management, New Taipei City 25245, Taiwan; 7 Graduate Institute of Long-Term Care, Mackay Medical College, New Taipei City 25245, Taiwan; 8 Graduate Institute of Biomedical Sciences, Mackay Medical College, New Taipei City 25245, Taiwan

**Keywords:** hematopoietic stress, osteoporosis, hemolytic anemia, national health insurance research database

## Abstract

Hemolytic anemia (HA) renders erythropoietic stress on the bone marrow and has been linked to osteoporosis. In this nationwide retrospective cohort study, we examined this correlation by utilizing the Taiwan National Health Insurance Research Database (NHIRD). We identified two cohorts, matching population with and without HA in a 1:4 ratio. A total of 2242 HA patients and 8968 non-HA patients were enrolled. Patients with HA had a significantly higher cumulative incidence (log-rank test *p* = 0.0073), higher incidence density (5.11 vs. 3.76 per 1000 persons-years), and a 1.31-fold risk of developing osteoporosis than non-HA patients (aHR = 1.31, 95% C.I. 1.04–1.63, *p* = 0.01). After adjusting for age, sex, and comorbidities, patients with factors including female (aHR = 2.57, 95% C.I. 2.05–3.22, *p* < 0.001), age > 65 (aHR = 9.25, 95% C.I. 7.46–11.50, *p* < 0.001), diagnosis of cholelithiasis (aHR = 1.76, 95% C.I. 1.20–2.58, *p* = 0.003) and peptic ulcer disease (aHR = 1.87, 95% C.I. 1.52–2.29, *p* < 0.001) had significantly higher risk of osteoporosis. We propose that this correlation may be related to increased hematopoietic stress, increased consumption of nitric oxide (NO) by hemolysis, and the inhibitory effects of iron supplements on osteogenesis through the receptor activator of nuclear factor κB ligand (RANKL)/Osteoprotegerin pathway and the Runt-related transcription factor 2 (RUNX2) factor. Our findings suggest that patients with hemolytic anemia are at a higher risk of developing osteoporosis, and it would be in the patient’s best interest for physicians to be aware of this potential complication and offer preventative measures.

## 1. Introduction

Osteoporosis is a chronic, progressive skeletal disease characterized by low bone density and microarchitectural defects in bone tissue. Osteoporosis is a major public health issue that is often overlooked and undertreated, resulting in a tremendous amount of resources allocated to potentially avoidable complications from increased bone fragility and susceptibility to fracture. In a study, only 25.6% of patients with fragility fractures were aware that they had osteoporosis, and 21.4% of them had been receiving osteoporosis treatment [1]. Osteoporosis has been more commonly associated in elderly, Caucasian women while common sites of fractures are hips, spine, and wrist, leading to enormous health and economic impacts, most notably in hip fractures [2]. Bone is an active tissue that is constantly remodeling in response to mechanical stresses and hormonal changes. This process is mediated by two types of cells dependent on each other for production and remodeling. Osteoclasts, derived from hematopoietic precursors, are responsible for bone resorption, whereas osteoblasts, originated from mesenchymal cells, are responsible for bone formation [3]. The imbalance in bone turnover, namely a relatively increased rate of bone absorption by osteoclast that exceeds the rate of bone formation by osteoblast, promotes loss of bone mass and incidence of osteoporosis, especially in postmenopausal women [4]. Annually, 2 million fractures due to osteoporosis occur in the United States and the cost paid by Medicare is expected to increase to $25 billion by 2025 as the population ages [5].

Hemolytic anemia encompasses several conditions that result in the premature destruction of red blood cells (RBCs). The causes can be categorized as hereditary and acquired, encompassing RBC membrane disorders, RBC enzyme defects, hemoglobinopathies, autoantibodies, medications, infection, hypersplenism, and malignancy. Sickle cell disease (SCD) and thalassemia are two common causes of the hereditary diseases. SCD is caused by an autosomal-recessive single gene defect in the beta chain of hemoglobin, which results in sickle cell hemoglobin (HbS). Sickle cells can be broken down prematurely. Thalassemia is the most common hemoglobinopathy, especially in malaria-endemic regions such as the Mediterranean, Middle East, northern Africa, and almost all of Southeast Asia. Thalassemia refers to a group of genetic disorders that reduce the production of the alpha or beta globin chains used for assembly of the hemoglobin molecule. The imbalance between alpha and beta chains leads to membrane damage and cell destruction. Warm-reactive autoimmune hemolytic anemia (AIHA) is the most common autoimmune-related anemia and mostly affects women. It may arise spontaneously or can be associated with diseases such as SLE, lymphoma, and chronic lymphocytic leukemia, among others [6,7]. Glucocorticoids are the first-line therapies for warm-reactive AIHA, although long-term use of glucocorticoids is associated with osteoporosis and osteonecrosis of the femoral head [8,9].

Sickle cell anemia, resulting from the homozygous S mutation (SS) of hemoglobin, is the most common phenotype of sickle cell disease [10]. It is well known that SCD is related to osteoporosis and bone infarction due to thrombosis of the end-arterial vessels in long-term chronic complications [11]. To a greater extent, skeletal abnormalities are found in patients with thalassemia, contributed by expansion and invasion of erythroid bone marrow, which widen the marrow spaces, attenuate the cortex, and therefore resulting in osteoporosis. However, there is no study investigating the relationship between osteoporosis and a specific hematological condition such as hemolytic anemia that can be observed among SCD, thalassemia, and AIHA patients. In this study, we aim to conduct the first nationwide retrospective study to investigate the risk of osteoporosis in patients with hemolytic anemia.

In this population-based retrospective cohort study, we aim to validate our hypothesis of an elevated risk of osteoporosis in patients with hemolytic anemia through data from Taiwan National Health Insurance Research Database (NHIRD). Other related factors, including sex, age, comorbidity, and iron supplementations were also analyzed.

## 2. Materials and Methods

### 2.1. Data Source

The Taiwan government established a nationwide database named the National Health Insurance Database (NHIRD), which contains the medical history, medication records, and other related records of insured people in Taiwan. As of today, more than 99% of Taiwan’s population is enrolled in the database. In the present study, we utilized the Longitudinal Health Insurance Database (LHID 2000) as the database to conduct the analyses. The LHID 2000 details have been described in previous studies [12,13,14]. It is composed of data obtained by randomly sampling 1 million subjects from NHIRD, retaining the sex and age distribution of the NHIRD, and undergoing the de-identification process of each subject. The diagnoses in Taiwan National Health Insurance are defined according to the International Classification of Disease, Ninth Revision, Clinical Modification (ICD-9-CM). The Ethics Review Board of China Medical University Hospital (CMUH-104-REC2-115) and the Institutional Review Board of MacKay Memorial Hospital (16MMHIS074) approved the study.

### 2.2. Study Population

In this study, we firstly identified two cohorts: HA cohort (ICD-9-CM 282) and non-HA cohort. The HA cohort was selected as patients with newly diagnosed HA during 2000–2007 and the diagnosed date was set as the index date. To achieve statistical power and control possible confounding factors, the comparison group was patients without HA and matched with HA cohort in a 1:4 ratio by sex, age, and index year. Patients with osteoporosis (ICD-9-CM 733.0) before the index date and less than 18 years old were excluded. The study period was set from the index date leading up to the timestamp of diagnosis with osteoporosis, withdrawal from the database, or, ultimately, until the end of 31 December 2013.

Comorbidities were important confounding factors in this study. We defined comorbidities as occurring before the index date and included inflammatory bowel disease (ICD-9-CM 555 and 556) [15], diabetes (ICD-9-CM 250) [7], cholelithiasis (ICD-9-CM 574), and peptic ulcer disease (ICD-9-CM 533) [16].

### 2.3. Statistical Analysis

To conduct the analysis of this study, the demographic factors and comorbidities were described in numbers and percentages, and the mean age was presented in mean and standard deviation. To demonstrate the difference of variables, chi-square test and t-test were used for categorical and continuous variables, respectively. The risk factors of osteoporosis in HA cohorts and non-HA cohorts were calculated by the Cox proportional hazard model and the risk was shown by hazard ratio (HR), adjusted hazard ratio (aHR), and 95% confidence interval (95% C.I.). The difference between cumulative incidence curves in HA and non-HA cohorts was calculated by the log-rank test. All statistical analyses were performed by SAS statistical software, version 9.4 (SAS Institute Inc., Cary, NC, USA). The figure of cumulative incidence curve was plotted by R software. The significance criterion was set at two-sided *p*-values less than 0.05 and the null value outside the 95% confidence interval.

## 3. Results

**Figure 1:**
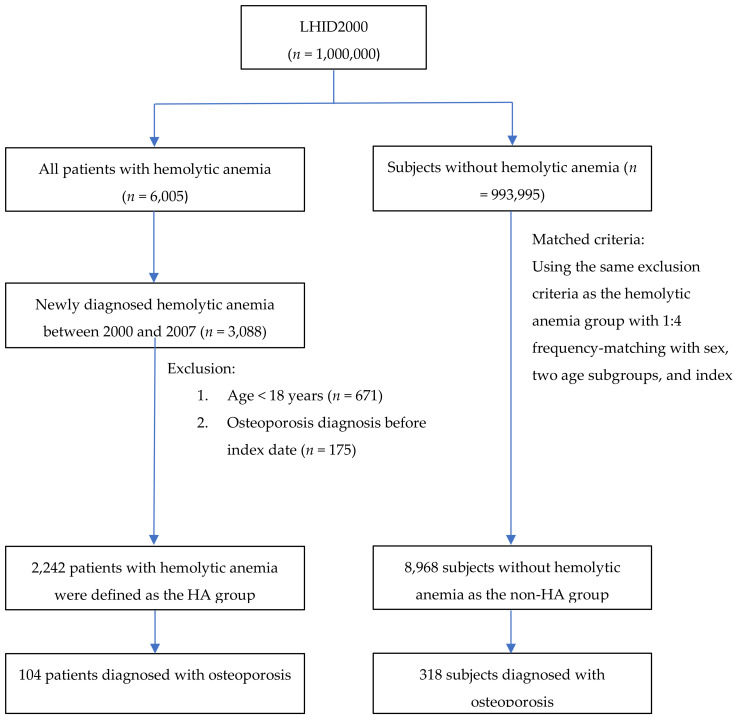
The selection process of the participants in the cohort study. Abbreviations: HA, hemolytic anemia; LHID, Longitudinal Health Insurance Database.

In order to investigate the association between HA and osteoporosis, we identified the demographic factors and comorbidities of study patients (Table 1). Of the 1,000,000 patients in the LHID2000 database, 6005 patients were diagnosed with hemolytic anemia. Among these patients, 3088 patients were newly diagnosed with osteoporosis during the study period; 671 patients were excluded due to young age (<18 years old) and 175 were excluded due to diagnosis of osteoporosis before the index date. In total, 11,210 study subjects were enrolled, including 2242 HA patients and 8968 non-HA patients (Figure 1). In this study, 63.3% were female, mean age was 39.3 years old. HA patients had a significantly higher percentage of inflammatory bowel disease (*p* = 0.04), diabetes (*p* < 0.001), cholelithiasis (*p* < 0.001), and peptic ulcer disease (*p* < 0.001).

**Figure 2:**
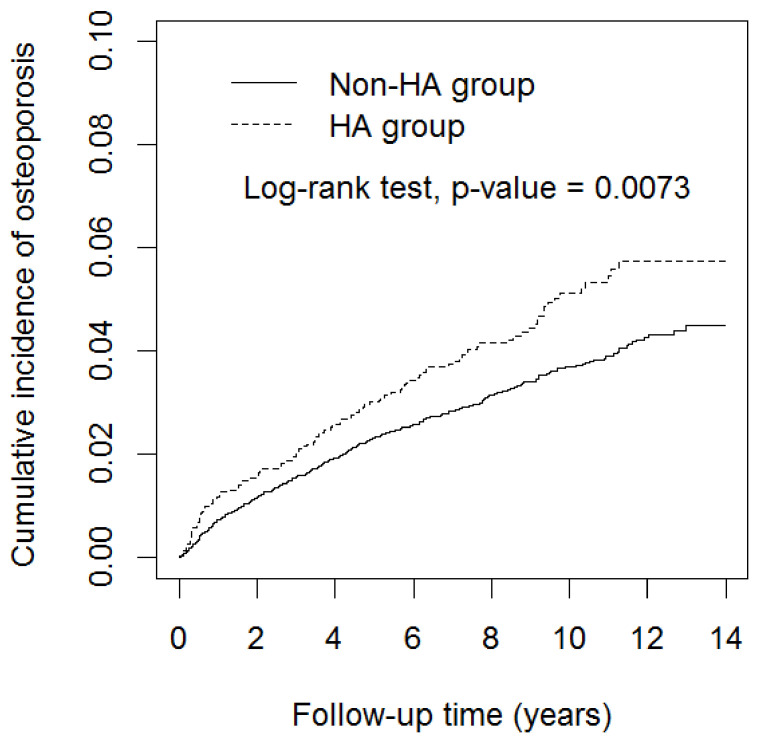
Cumulative incidence curves of osteoporosis for groups with and without hemolytic anemia. Abbreviation: HA, hemolytic anemia.

The results of the cohort study (Figure 2) showed that patients with HA had a significantly higher cumulative incidence of osteoporosis than non-HA group (log-rank test *p* = 0.0073).

The incidence densities of osteoporosis in HA patients and non-HA patients were 5.11 and 3.76 (per 1000 person-years), respectively, and HA patients had a 1.31-fold risk of developing osteoporosis than non-HA patients (aHR = 1.31, 95% C.I. 1.04–1.63, *p* = 0.01), as shown in Table 2. After adjusting for age, sex, and comorbidities, we found that women (aHR = 2.57, 95% C.I. 2.05–3.22, *p* < 0.001), age > 65 years old (aHR = 9.25, 95% C.I. 7.46–11.50, *p* < 0.001), patients with cholelithiasis (aHR = 1.76, 95% C.I. 1.20–2.58, *p* = 0.003), and peptic ulcer disease (aHR = 1.87, 95% C.I. 1.52–2.29, *p* < 0.001) had a significantly higher risk of osteoporosis.

Further stratification (Table 3) also showed that the HA group of women (aHR = 1.68, 95% C.I. 1.31–2.16, *p* < 0.001), aged between 20 and 64 years old (aHR = 1.53, 95% C.I. 1.15–2.02, *p* = 0.003), and patients without comorbidities (aHR = 1.57, 95% C.I. 1.11–2.22, *p* = 0.01) had significantly higher risk of osteoporosis as compared with the non-HA group.

We further classified HA groups into subgroups: HA patients with or without iron supplements and HA patients with or without blood transfusion and compared them with the non-HA group (Table 4). The results showed that HA patients on iron supplementations (aHR = 1.34, 95% C.I. 1.01–1.79, *p* = 0.04) or not on iron supplements (aHR = 1.51, 95% C.I. 1.11–2.05, *p* = 0.008), and patients without blood transfusion (aHR = 1.49, 95% C.I. 1.19–1.86, *p* < 0.001) had a significantly higher risk of developing osteoporosis after adjusting for demographic factors and comorbidities. Iron supplements for hemolytic anemia may indicate the intrinsic factors that affect the incidence of osteoporosis [17,18]. Therefore, pharmacological treatments for hemolytic anemia may have a major role in increasing the risk of osteoporosis whereas blood transfusions may play a protective role.

## 4. Discussion

In the present article, we observed the increased risk of osteoporosis in patients with hemolytic anemia via the Taiwan National Health Insurance Research Database (NHIRD). Women, the elderly, and cholelithiasis or peptic ulcer disease patients had higher risk of osteoporosis after the HRs were adjusted for age, sex, and comorbidities. Compared with the non-HA group, the HA group of women, aged between 20 and 64 years old, and patients without comorbidities had higher risk of osteoporosis. In terms of iron supplements and blood transfusion for HA patients, only patients receiving blood transfusion therapy had a lower risk of osteoporosis.

Our study showed that women and elderly subjects had a significantly higher risk of osteoporosis. Deficiency of estrogen was reported to promote hypoxia inducible factor 1 alpha (HIF1α) protein, which was identified as essential for osteoclast activation [19]. The World Health Organization (WHO) defines osteoporosis in postmenopausal women as a bone mineral density (BMD) value at the spine, hip, or forearm of 2.5 or more standard deviations (SD) below the young adult mean (T-score ≤ −2.5), with or without the presence of a fragility fracture [20]. Early diagnosis and quantification of bone loss and fracture risk are important because of the availability of therapies, including lifestyle modifications, which can slow or even reverse the progression of osteoporosis. Alendronic acid, risedronate, etidronate, and strontium ranelate are treatment options for the primary prevention of osteoporotic fragility fractures in postmenopausal women [21].

The risk of osteoporosis was higher in patients with cholelithiasis and peptic ulcer disease (Table 2). Despite the lack of robust evidence, the population-based study implies osteoporosis has a higher risk of gallstone development [22]. A large cohort study disclosed that the risk of osteoporosis was 1.85 times higher in the peptic ulcer disease (PUD) group compared to the non-PUD group, since the usage of proton pump inhibitors (PPIs) was indicated to increase subsequent risk of osteoporosis [23]. In contrast, PUD may result in dietary restrictions that cause osteoporosis by way of insufficient calcium intake [24].

Compared with the non-HA group, women, aged between 20 and 64 years old and patients without comorbidities in the HA group had significantly higher risk of osteoporosis (Table 3). Low bone mass density (BMD) was indicated to be associated with hemolysis (high lactate dehydrogenase, high reticulocyte count, and low hemoglobin) [25].

Moreover, in Table 4, HA patients receiving blood transfusions have lower risk of osteoporosis compared with those who had not received blood products (aHR = 1.49, 95% C.I. 1.19–1.86, *p* < 0.001). This result is compatible with previous studies that suggest low hemoglobin levels lead to low bone mass density [26]. The result also showed that HA patients on medical therapy had a significantly higher risk of osteoporosis. Immune suppressors, one of the treatments for autoimmune hemolytic anemia (AIHA), such as corticosteroid, rituximab, and cyclosporine, often induce the occurrence of osteoporosis. A daily dose of ≥5 mg prednisolone for over 3 months has been shown to result in increasing risk of fractures [27]. Thus, the finding of our current study illustrates that both blood transfusion and oral drugs play potential roles in the occurrence of osteoporosis, although further mechanisms would need to be explored in future studies.

Iron supplementations and blood transfusions are two of the most common treatments to raise hemoglobin levels. For patients with hemolytic anemia, treatments differ according to etiologies. For example, the main management of sickle cell anemia was blood transfusion to reduce the percentage of HbS in the serum [28,29]. In terms of thalassemia—one of the most major etiology of HA, blood transfusion, is the mainstay therapy for beta thalassemia major. It is also an essential treatment for beta thalassemia intermedia and alpha thalassemia intermedia if hemoglobin becomes inadequate or hemolysis occurs [30]. Thalassemic bone complications were reported in thalassemia major or transfusion-dependent thalassemia populations [31]. One cohort study revealed a 1.35-fold increased risk of fractures in transfusion-naïve thalassemia population when compared with non-thalassemia controls [32,33].

**Figure 3:**
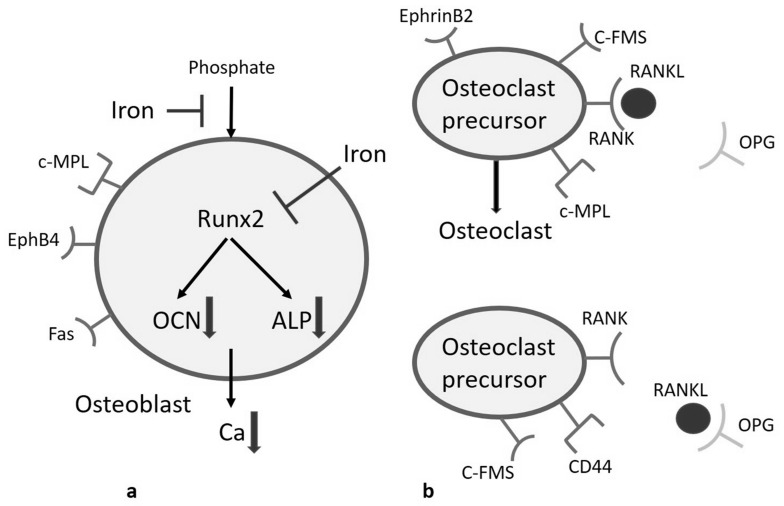
(**a**) Iron decreases osteoblast activity by inhibiting phosphate uptake and downregulating RUNX2. (**b**) OPG plays a role in a decoy receptor for RANKL to inhibit osteoclastogenesis. Abbreviations: RUNX2, Runt-related transcription factor 2; OCN, osteocalcin; ALP, alkaline phosphatase; RANKL, receptor activator of nuclear factor κB ligand; OPG, osteoprotegerin.

Iron is required for the formation of hemoglobin, myoglobin, and heme enzymes, so iron deficiency leads to impaired hemoglobin synthesis, resulting in hypochromic, microcytic anemia. In contrast, excess iron can lead to tissue damage, inflammation, and fibrosis, mostly associated in liver, heart, joints, skin, and endocrine organs [34]. Iron decreases phosphate uptake and downregulates Runt-related transcription factor 2 (RUNX2), an osteoblast-specific transcription factor in vitro study. Its target transcripts, osteocalcin (OCN) and alkaline phosphatase (ALP), are inhibited by suppression of RUNX2. With decreased osteoblast activity, calcium deposition decreases as well (Figure 3a) [30,35,36]. Several studies also indicate that serum iron will induce cleaved caspase 3 and caspase 7 of osteoblasts, inducing cell apoptosis [37]. Furthermore, oxidative stress (e.g., H_2_O_2_, ROS) produced by iron can inhibit osteoblast activity. Iron-containing compounds such as Hb or hemin that are released during hemolysis give rise to oxidative stress as well [38]. The crucial transcription pathways for osteoblastogenesis, including RUNX2, Wnt-βcatenin, and ERK, are regulated by oxidative stress [39]. In one dose-dependent manner in vitro study, ferric (III) iron and ferrous (II) iron both decreased osteoblast cell survival rate, but osteoblast was more sensitive to ferric (III) than ferrous (II) [37]. However, many studies have pointed out that iron promotes osteoclast differentiation, activation, and bone resorption. Receptor activator of nuclear factor κB ligand (RANKL) and osteoprotegerin (OPG) regulate osteoclast differentiation. While RANKL stimulates osteoclastogenesis, OPG plays a role as a decoy receptor for RANKL to inhibit osteoclastogenesis (Figure 3b) [40]. Both in vitro and in vivo studies indicated iron overload elevates RANKL/OPG ratio to promote osteoclast activity and bone resorption [41].

Blood transfusion will increase concentration of ferrous (II) iron by supplementing hemoglobin. In other words, the patient increases intestinal iron uptake and serum ferric (III) concentration sequentially rises without blood transfusion [42]. Ferric (III) iron increases by iron supplements intake, but ferrous (II) iron increases by blood transfusion therapy in thalassemia patients [37]. These phenomena are consistent with our finding that the iron supplements group and the nonblood transfusion therapy group have higher incidences of osteoporosis by increasing serum ferric (III) iron (Table 4). The balance between ferric (III) and ferrous (II) iron related to osteoporosis should be investigated in further studies.

Our study has several strengths. First, this is the first nationwide retrospective study investigating the association of osteoporosis and hemolytic anemia. Based on this study, other researchers can advance the topics by different study designs. Second, this study can help healthcare providers prevent patients with hemolytic anemia or comorbidities from osteoporosis and low-trauma fractures. Hospital managers and physicians can identify the patients at high risk of hospital fall-related injury as early as possible. With appropriate surveillance and prevention, healthcare providers can minimize hospitalized patients’ physical and economic burden, including medical cost, lengths of stay, quality of life, and mortality rate. Conversely, our study has some limitations. First, the NHIRD did not include laboratory data, lifestyle, exercise capacity, body weight, nutrition supplements, or family histories of chronic diseases, so not all factors were considered to measure risk of osteoporosis. However, we already accounted for these related factors by using comorbidities. Second, the population studied was mainly composed of Southeast Asians living in Taiwan, so whether ethnic or geographic discrepancies exist in different populations might require further studies. Nevertheless, in our study, the diagnosis of osteoporosis and others were based on the ICD-9-CM codes, as determined by qualified clinical physicians for the strictly audited reimbursement process. Furthermore, NHIRD covers a highly representative sample of Taiwan’s general population because the reimbursement policy is universal and operated by a single-buyer, the Taiwanese government. All insurance claims are scrutinized and coded by medical reimbursement specialists and peer reviewed according to the standard criteria for diagnoses in the study. Moreover, incorrect diagnoses or coding mistakes result in considerable penalties for physicians. The reliability and validity of the NHI research database for epidemiologic investigations have been reported previously. Therefore, the diagnoses and coding in this study should be highly reliable. Third, we did not investigate the effect of drugs given for hemolytic anemia or osteoporosis, such as calcium or vitamin D3 supplements, or glucocorticoids, and they may interfere with the development of osteoporosis. It is worth conducting further studies to investigate the role of drug use between hemolytic anemia and osteoporosis.

## 5. Conclusions

In summary, the clinical implications of our study highlight the need for hemolytic anemia patients and their physicians to be aware of the subsequent risk of osteoporosis. Appropriate preventive measurements could be taken to have a profound improvement in quality of life of patients with hemolytic anemia.

## Figures and Tables

**Table 1:** Demographic factors and comorbidity of study participants according to hemolytic anemia status.

	HA Group *n* = 2,242		Non- HA Group *n* = 8,968		*p*-Value
Variable	*n*	%	*n*	%	
Sex					>0.99
Female	1,419	63.3	5,676	63.3	
Male	823	36.7	3,292	36.7	
Age, years					>0.99
20–64	2,019	90.1	8,076	90.1	
≥65	223	9.95	892	9.95	
Means (SD) ^a^	39.3	(16.3)	39.3	(16.3)	0.99
Comorbidity					
Inflammatory bowel disease	43	1.92	120	1.34	0.04
Diabetes	177	7.89	418	4.66	<0.001
Cholelithiasis	85	3.79	160	1.78	<0.001
Peptic ulcer disease	702	31.3	1,928	21.5	<0.001

Abbreviations: HA, hemolytic anemia; SD, standard deviation. ^a^ Student’s *t*-test.

**Table 2:** Cox model-measured hazard ratios and 95% confidence interval of osteoporosis associated with hemolytic anemia and covariates.

Variable	Event No.	Person-Years	Incidence Density ^a^	HR (95% C.I.)			
				Unadjusted	*p*-Value	Adjusted ^b^	*p*-Value
Hemolytic anemia							
No	318	84,474	3.76	ref		ref	
Yes	104	20,355	5.11	1.35 (1.08–1.69)	0.007	1.31 (1.04–1.63)	0.01
Sex							
Female	315	66,488	4.74	1.70 (1.37–2.12)	<0.001	2.57 (2.05–3.22)	<0.001
Male	107	38,340	2.79	ref		ref	
Age, years							
20–64	238	97,295	2.45	ref			
≥65	184	7,534	24.4	9.54 (7.86–11.6)	<0.001	9.25 (7.46–11.5)	<0.001
Comorbidity							
IBD							
No	409	103,456	3.95	ref		ref	
Yes	13	1,372	9.47	2.34 (1.34–4.06)	0.002	1.47 (0.84–2.57)	0.17
Diabetes							
No	363	100,505	3.61	ref		ref	
Yes	59	4,324	13.7	3.60 (2.74–4.75)	<0.001	1.24 (0.92–1.67)	0.15
Cholelithiasis							
No	391	103,032	3.79	ref		ref	
Yes	31	1,796	17.3	4.35 (3.01–6.27)	<0.001	1.76 (1.20–2.58)	0.003
Peptic ulcer disease							
No	226	81,736	2.77	ref		ref	
Yes	196	23,093	8.49	3.02 (2.50–3.66)	<0.001	1.87 (1.52–2.29)	<0.001

Abbreviations: HA, hemolytic anemia; HR, hazard ratio; CI, confidence interval; IBD, inflammatory bowel disease. ^a^ per 1000 person-years. ^b^ Multivariate Cox proportional hazards regression model including hemolytic anemia, sex, age, inflammatory bowel disease, diabetes, cholelithiasis, and peptic ulcer disease.

**Table 3:** Incidence density and hazard ratios of osteoporosis according to hemolytic anemia status stratified by sex, age, and comorbidity.

	HA Group			Non-HA Group			HR (95% C.I.)			
Variables	Event No.	Person-Year	Incidence Density ^a^	Event No.	Person-Year	Incidence Density ^a^	Crude	*p*-Value	Adjusted ^b^	*p*-Value
Sex										
Female	87	12,905	6.74	228	53,583	4.26	1.58 (1.23–2.02)	<0.001	1.68 (1.31–2.16)	<0.001
Male	17	7,449	2.28	90	30,891	2.91	0.78 (0.47–1.32)	0.35	0.77 (0.46–1.30)	0.33
*p* for interaction										0.02
Age, years										
20–64	72	19,089	3.77	166	78,206	2.12	1.77 (1.35–2.34)	<0.001	1.53 (1.15–2.02)	0.003
≥65	32	1,266	25.3	152	6,268	24.3	1.01 (0.69–1.49)	0.94	0.99 (0.67–1.45)	0.94
*p* for interaction										0.04
Comorbidity status ^c^										
No	41	13,278	3.09	145	64,747	2.24	1.38 (0.98–1.95)	0.06	1.57 (1.11–2.22)	0.01
Yes	63	7,077	8.90	173	19,727	8.77	1.02 (0.76–1.36)	0.91	1.27 (0.95–1.69)	0.11
*p* for interaction										0.18

Abbreviations: HA, hemolytic anemia; HR, hazard ratio; CI, confidence interval. ^a^ per 1000 person-years. ^b^ Model mutually adjusted for sex, age, and each comorbidity (including inflammatory bowel disease, diabetes, cholelithiasis, and peptic ulcer disease). ^c^ Patients with any one of inflammatory bowel disease, diabetes, cholelithiasis, and peptic ulcer disease were classified as the comorbidity group.

**Table 4:** Incidence density and hazard ratios of osteoporosis for hemolytic anemia status combined with therapy.

Variables	*n*	Event No.	Person-Year	Incidence Density ^a^	HR (95% C.I.)			
					Crude	*p*-Value	Adjusted ^b^	*p*-Value
Non-HA group	8,968	318	84,474	3.76	ref		ref	
HA group								
Iron supplements								
No	1,498	56	13,476	4.16	1.10 (0.83–1.46)	0.51	1.34 (1.01–1.79)	0.04
Yes	744	48	6,878	6.98	1.85 (1.36–2.50)	<0.001	1.51 (1.11–2.05)	0.008
Blood transfusion								
No	2,169	102	19,764	5.16	1.37 (1.09–1.71)	0.006	1.49 (1.19–1.86)	<0.001
Yes	73	2	591	3.39	0.88 (0.22–3.53)	0.86	0.41 (0.10–1.66)	0.21

Abbreviations: HA, hemolytic anemia; HR, hazard ratio; CI, confidence interval. ^a^ per 1000 person-years. ^b^ Model adjusted for sex, age, inflammatory bowel disease, diabetes, cholelithiasis, and peptic ulcer disease.

## Data Availability

The data underlying this article were provided by the National Health Insurance Research Database (NHIRD) by permission. Data will be shared on request to the corresponding author with permission of the National Health Insurance Research Database.
